# EuroPineDB: a high-coverage web database for maritime pine transcriptome

**DOI:** 10.1186/1471-2164-12-366

**Published:** 2011-07-15

**Authors:** Noé Fernández-Pozo, Javier Canales, Darío Guerrero-Fernández, David P Villalobos, Sara M Díaz-Moreno, Rocío Bautista, Arantxa Flores-Monterroso, M Ángeles Guevara, Pedro Perdiguero, Carmen Collada, M Teresa Cervera, Álvaro Soto, Ricardo Ordás, Francisco R Cantón, Concepción Avila, Francisco M Cánovas, M Gonzalo Claros

**Affiliations:** 1 Departamento de Biología Molecular y Bioquímica, Facultad de Ciencias, Campus de Teatinos s/n, Universidad de Málaga, 29071 Málaga, Spain; 2 Plataforma Andaluza de Bioinformática, Edificio de Bioinnovación, C/Severo Ochoa 34, Universidad de Málaga, 29590 Málaga, Spain; 3 Departamento de Ecología y Genética Forestal, CIFOR-UNIA, Carretera de La Coruña, km 7,5, 28040 Madrid, Spain; 4 UM Genómica y Ecofisiología Forestal INIA-UPM, Universidad Politécnica de Madrid, Madrid, Spain; 5 Área de Fisiología Vegetal, Departamento BOS, Instituto Universitario de Biotecnología de Asturias, Universidad de Oviedo, 33071 Oviedo, Spain

## Abstract

**Background:**

*Pinus pinaster *is an economically and ecologically important species that is becoming a woody gymnosperm model. Its enormous genome size makes whole-genome sequencing approaches are hard to apply. Therefore, the expressed portion of the genome has to be characterised and the results and annotations have to be stored in dedicated databases.

**Description:**

EuroPineDB is the largest sequence collection available for a single pine species, *Pinus pinaster *(maritime pine), since it comprises 951 641 raw sequence reads obtained from non-normalised cDNA libraries and high-throughput sequencing from adult (xylem, phloem, roots, stem, needles, cones, strobili) and embryonic (germinated embryos, buds, callus) maritime pine tissues. Using open-source tools, sequences were optimally pre-processed, assembled, and extensively annotated (GO, EC and KEGG terms, descriptions, SNPs, SSRs, ORFs and InterPro codes). As a result, a 10.5× *P. pinaster *genome was covered and assembled in 55 322 UniGenes. A total of 32 919 (59.5%) of *P. pinaster *UniGenes were annotated with at least one description, revealing at least 18 466 different genes. The complete database, which is designed to be scalable, maintainable, and expandable, is freely available at: http://www.scbi.uma.es/pindb/. It can be retrieved by gene libraries, pine species, annotations, UniGenes and microarrays (i.e., the sequences are distributed in two-colour microarrays; this is the only conifer database that provides this information) and will be periodically updated. Small assemblies can be viewed using a dedicated visualisation tool that connects them with SNPs. Any sequence or annotation set shown on-screen can be downloaded. Retrieval mechanisms for sequences and gene annotations are provided.

**Conclusions:**

The EuroPineDB with its integrated information can be used to reveal new knowledge, offers an easy-to-use collection of information to directly support experimental work (including microarray hybridisation), and provides deeper knowledge on the maritime pine transcriptome.

## 1 Background

Conifers (*Coniferales*), the most important group of gymnosperms, represent 650 species, some of which are the largest, tallest, and oldest non-clonal terrestrial organisms on Earth. They are of immense ecological importance, dominating many terrestrial landscapes and representing the largest terrestrial carbon sink. Currently present in a large number of ecosystems, they have evolved very efficient physiological adaptation systems. Given that trees are the great majority of conifers, they provide a different perspective on plant genome biology and evolution taking into account that conifers are separated from angiosperms by more than 300 million years of independent evolution. Studies on the conifer genome are revealing unique information which cannot be inferred from currently sequenced angiosperm genomes (such as poplar, *Eucaliptus*, *Arabidopsis *or rice): around 30% of conifer genes have little or no sequence similarity to plant genes of known function [1,2]. Unfortunately, conifer genomics is hindered by the very large genome (e.g. the pine genome is approximately 160 times larger than *Arabidopsis *and seven times larger than the human genome; in fact, it is larger than any other genome sequenced to date) that is replete with highly repetitive, non-coding sequences [3].

Conifers include the economically and ecologically important species of spruces (*Picea sp*) and pines (*Pinus*), *Pinus *being the largest extant genus with approximately 115 species. The importance of pines is due to the fact that: (i) their timber and paper pulp are used for the construction of buildings and furniture; (ii) they are used in reforestation due to their rapid growth and drought tolerance as compared to other tree species; (iii) they help stabilise sandy soils and indirectly act as an atmospheric CO_2 _sink, helping to reduce global warming; (iv) some pine nuts are widely used in Mediterranean cuisine. Consequently, the genus *Pinus *is becoming a woody gymnosperm model. The main pine model species in Europe are *Pinus pinaster *and *Pinus sylvestris*, whereas *Pinus taeda *and *Pinus contorta *are the equivalent in North America. Therefore, it is relevant to investigate and increase our knowledge of the content of the pine genome as this would allow the exploitation of natural genetic resources and the use of new forest reproductive material appropriate to adapt these trees to a changing climate.

The application of genome-based science is playing an important role in understanding the genome content and structure of different organisms. Since whole-genome sequencing approaches are hard to apply to large genomes such as the pine genome, scientists have focused on the expressed portion of the genome using dedicated technologies. For example, the sequencing of clones obtained by suppression subtractive hybridisation (SSH) [4-6] provides gene-enriched sequences that are specific to a particular condition. However, the dominant approach to characterising the transcriptionally active portions of pine genomes has been expressed sequence tags (ESTs) [7,8] due to the absence of non-coding DNA (mainly introns and intergenic regions). Classic ESTs are subject to artefacts during cDNA library construction and are highly error prone during sequencing procedures. As a result, erroneous clustering and assembling occur during reconstruction of putative transcripts and may ultimately lead to inaccurate gene annotation [9]. However, next-generation sequencing technologies have removed many drawbacks and time-consuming steps involved in classic ESTs and have facilitated transcriptome sequencing of many species at a fraction of the total time and cost previously required [10]. ESTs have also driven the development of pine microarrays [11-14], although there is no easy way to relate the data printed on these microarrays to the corresponding pine sequences.

Sequencing projects should store, organise, and retrieve sequences by means of user-friendly databases. Since many sequences in EST databases are reported to be highly contaminated or incorrectly pre-processed [9], there is a need for more reliable pre-processing, clustering, assembly and annotation pipelines to yield reliable information. ConiferEST [15] (now part of ConiferGDB http://www.conifergdb.org/coniferEST.php) was the first attempt to rationalise pine sequences by more precise pre-processing dedicated to *Pinus taeda *traces only. The DFCI Pine Gene Index http://compbio.dfci.harvard.edu/cgi-bin/tgi/gimain.pl?gudb=pine, a subset of the discontinued TGI Gene Indices [16], is a non-redundant database of all putative *Pinus *genes. This is a very large compilation of pine sequences, but only GO and KEGG annotations are available and no separation by species is provided, *P. taeda *is highly over-represented, and its interface only allows limited interaction. ForestTreeDB was created to centralise large-scale ESTs from diverse tissues of conifer and poplar trees [1], but it is no longer available. The TreeGenes database http://dendrome.ucdavis.edu/treegenes/ is composed of a wide range of forest tree species [17]. This effort to combine and inter-relate a great variety of different information should be acknowledged, even though EST pre-processing is not optimal. TreeSNPs [18] and PineSAP [19] are databases exclusively devoted to single nucleotide polymorphisms (SNPs) in *Picea *and *Pinus *species, respectively. Recently, Parchman and co-workers [2] described the first high-throughput analysis of a pine species, but no database was created for this. It should be noted that none of the above databases are linked to the pine microarrays described in literature.

Our group has been working on pine genomics for many years (e.g., EMBL accession numbers AM982822-AM983454, BX248593-BX255804, BX682240-BX683073, BX784033-BX784385, EC428477-EC428747, FM945441-FM945999 or FN256437-FN257130) and wish to provide high-quality sequences and annotations of pine genomes by means of EuroPineDB. Taking advantage of next-generation sequencing methods, recently released pre-processors [20], reliable sequence annotators [21], and the bioinformatics infrastructure of the University of Málaga (Spain), EuroPineDB was designed to gather the most reliable re-pre-processed, assembled, and annotated *P. pinaster *sequences using different technologies. Retrieval systems based on sequence similarity, description matches or microarray positions are also included, as well as browsing by species, experimental process, and annotation. As a new feature, many of its sequences have been printed on a microarray for expression analysis [22] and can be freely browsed.

## 2 Construction and content

### 2.1 Pine sequences

Although EuroPineDB is mainly devoted to the *P. pinaster *(maritime pine) genome, several sequences from two other species (*P. sylvestris *and *P. pinea*) are also included since their sequences were printed in a pine microarray (see below).

#### 2.1.1 Gene libraries

**Figure 1:**
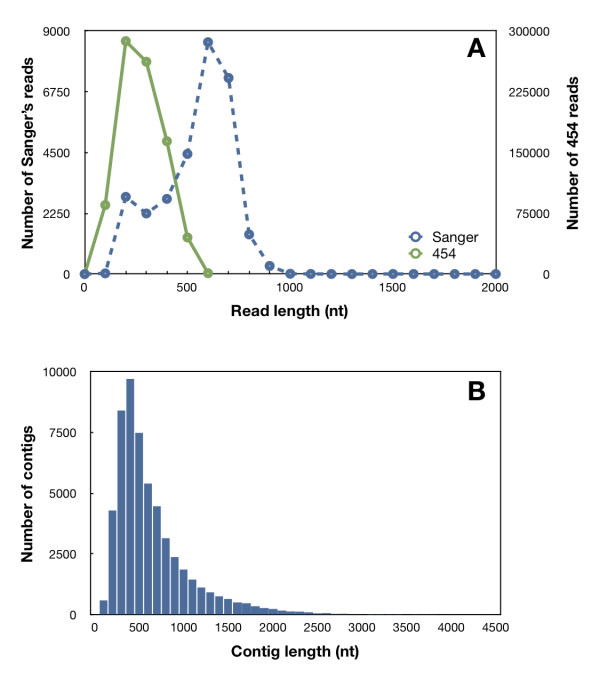
**A, Size distribution of pre-processed 454 and Sanger's reads used for EuroPineDB**. As expected, Sanger reads were longer than 454 reads in length. **B**, Contig size distribution within EuroPineDB.

Different gene libraries, all of which were constructed using different tissues and different strategies (described in Table 1) were included. All libraries were sequenced using Sanger's dideoxynucleotide method except for the sequences generated from Pp-454, which were obtained with a GS-FLX pyrosequencer using Titanium technology. Pp-454 was the main contributor to the database (55 431 UniGenes and 844 737 curated reads). Frequency distributions of reads and contigs are shown in Figure 1.

**Table 1:** Gene libraries providing sequences for EuroPineDB

Gene library	Tissue	Species	Experimental conditions
Pp-454	Roots, stem, embryos, callus, cones, male and female strobili, buds, xylem, phloem.	*P. pinaster*	ESTs from several different tissues
LG0BCA	Buds	*P. pinaster*	ESTs, adult buds
GEMINI^a^	Xylem	*P. pinaster*	ESTs from normal, compression, opposite, early and late wood
SSH Xylem	Xylem	*P. pinaster*	SSH, compression vs. opposite, and juvenile vs. mature
UPM	Roots, stem, needles	*P. pinaster*	SSH, drought stress
ARG	Roots	*P. pinaster*	SSH, ammonium excess vs. ammonium deficiency
SSH Lac-Pine	Roots	*P. pinaster*	SSH, inoculated with *Laccaria bicolor *vs. not inoculated
SSH Mic	Roots	*P. pinaster*	SSH, mycorrhizal vs. not mycorrhizal
CK16^b^	Cotyledons	*P. pinea*	SSH, adventitious shoot induction
SSH Embryos	Embryos	*P. sylvestris*	SSH, lack of N vs. normal N
Pin	Cotyledons	*P. sylvestris*	ESTs from photosynthetic tissues
EMBL v. 102	-	*P. pinaster*, *P. pinea*, *P. sylvestris*	Miscellaneous

^a ^GEMINI gene library was described in [8]

^b ^CK16 gene library was described in [4]

#### 2.1.2 EMBL sequences

Gene library reads were completed with 13 206 sequences from the EMBL v. 102 database including the plant EST (Expressed Sequence Tag) and plant STD (Standard) sets for sequences whose 'source organism' field contained *P. pinaster, P. sylvestris *or *P. pinea*, provided that the sequence was not already included as a member of one of the gene libraries, and discarding any organellar sequence or sequences whose length was below 100 bp. The idea was to gather all sequence data on the three species, including their annotations. With *P. pinaster *as the main contributor (12 673 out of 13 206 EMBL entries), EMBL entries only provided 5667 different UniGenes.

#### 2.1.3 Microarray

Before EuroPineDB was constructed -- based on the existing putative UniGenes http://cbi.labri.fr/outils/SAM/COMPLETE/index.php?ID=gemini -- an EST-based microarray was designed containing 3456 spots printed twice with clones taken from the Pin, Gemini and CK16 gene libraries only (Table 2) [22]. Spots were distributed on the chip into 16 blocks of 16 × 14 dots. The microarray also included some full-length cDNA sequences of genes related to nitrogen metabolism, such as aspartate aminotransferase, asparagine synthetase, L-asparaginase, glutamine synthetase *a *and *b*, NADP^+ ^isocitrate dehydrogenase and ornithine aminotransferase, which can be found in the EMBL v102 set. The inclusion of microarray information in EuroPineDB facilitates accessing the most complete information on each sequence printed on it. In the near future, microarrays implemented with sequences contained in EuroPineDB will be also included.

**Table 2:** Statistics for the gene libraries shown in Table 1

Gene library	Raw	Curated	Mean **length**^***a***^	Singletons	Contigs	UniGenes (% annotated)	Discarded nt (%) by		
							
							QV	Vector	Artefacts*^b^*
Pp-454	913 786	844 737	227	471	54 960	55 431 (59.5%)	52.5%	NA	3.03%
LG0BCA	8766	8766	608	3834	1363	5197 (68.2%)	NA	NA	0.24%
GEMINI	13 057	7916	458	3066	1124	4190 (49.9%)	9.4%	10.4%	2.9%
SSH Xylem	992	790	474	385	142	527 (49.5%)	5.35%	31.8%	2.5%
UPM	2806	1115	465	258	157	415 (31.8%)	3.2%	15.9%	21.04%
ARG	218	148	394	127	7	134 (47.8%)	22.5%	5.1%	5.3%
SSH Lac-Pine	351	231	350	210	8	218 (34.4%)	18.5%	4.7%	2.64%
SSH Mic	294	194	314	149	13	162 (38.3%)	15.3%	13.4%	5.75%
CK16	358	282	575	221	24	245 (65.3%)	NA	0.05%	6.6%
SSH Embryos	96	57	437	34	6	40 (57.5%)	1.7%	20.6%	8.8%
Pin	863	617	532	335	86	421 (68.9%)	10.2%	9%	2.9%
EMBL v. 102	13 206	12 673	502	3704	1963	5667 (NA)	NA	0.1%	0.58%

TOTAL	954 793	880 295							

*P. pinaster*	951 641	877 523	597	684	54 648	55 332 (59.5%)			
*P. sylvestris*	2770	2466	730	476	203	679 (65.9%)			
*P. pinea*	382	306	574	239	27	266 (63.2%)			

QV, quality value. NA, not applicable.

*^a ^*Mean lengths are calculated with gene library reads. Nevertheless, they are calculated for contigs in the last three rows corresponding to the three species.

*^b ^*Artefacts include poly-A, poly-T, adaptors, contaminant sequences, and chimerical inserts.

### 2.2 Database architecture

The EuroPineDB was built using Ruby On Rails 2.0 http://rubyonrails.org/, a web development framework that uses a model-view-controller pattern to maintain strict separation between the web interface (views) code, database tables (models), and all methods that handle interactions between views and database (controllers). It also maintains different environments for each development phase (development, production and testing). This enabled EuroPineDB to be developed and tested in a redundant Oracle RAC (Real Application Cluster) database. Bulk imports, updates, and database managements were automated by means of Ruby scripts.

An automated pipeline that combines all the tools described here is executed on every EuroPineDB update. An update incorporates new pine sequence retrieval from dbEST and EMBL databases, new user reads, and the re-execution of bioinformatics tools with every new UniGene.

### 2.3 Pre-processing and assembling entries

Most problems for automated sequence assembly resulted from chimerical clones in the plasmid libraries, bacterial DNA contamination, low-quality sequences, and low-complexity repeats. Since ESTs in databases are commonly inaccurately pre-processed, any data sequence contained in EuroPineDB whose quality values (QV) for each nucleotide are available was pre-processed using SeqTrim http://www.scbi.uma.es/seqtrim with parameter customisation for each type of library [20]. High-quality pre-processed EST and SSH sequences guarantee that only non-chimerical, good-quality sequences (i.e., reads devoid of vectors, adaptors, poly-A/T tails, contaminants, and potential cloning artefacts) are included in the database, while sequences consisting of cloning vectors (AC BX676903) or containing poly-A (AC BX676940) were removed. The better the quality of the trimmed sequences, the more reliable the final assembly. It should be noted that 52.5% of nucleotides from the Pp-454 gene library were discarded due to low quality. The EMBL v. 102 subset included in EuroPineDB was also pre-processed with SeqTrim in order to remove uninformative, contaminant or erroneous sequences, even though these sequences lack a QV.

Curated sequences, i.e. those longer than 100 bp for Sanger's reads and 60 bp for 454 reads that exceed SeqTrim pre-processing, were assembled to produce putative pine transcriptional units (UniGenes) as contigs and singletons. Sanger's reads were *de novo *assembled with a web version of CAP3 [http://www.scbi.uma.es/cap3, [23]] since it has been described as a highly reliable sequence assembler for establishing UniGenes [24] with these types of reads. CAP3 assembly was conducted with default parameters using 85% as the cut-off for overlap percent identity to deal with the sequence variation due to the high heterozygosity of pine genes and genome heterogeneity between samples (Table 1). The 454 reads were *de novo *assembled with a web version of MIRA3 [http://www.scbi.uma.es/mira, [25]] using 454 settings for ESTs, which enormously reduces the number of misassembled contigs; moreover, this assembly contains 99.94% of curated reads and provides as few as 471 singletons (0.06%). Each library sequence set (Table 1) was assembled separately to obtain specific UniGene assemblies for every gene library and every pine species (Table 2); *P. pinaster *comprehensive assembly was also performed with MIRA3 with mixed 454-Sanger settings. A collection of all UniGenes is available for every assembly and the distribution of contig lengths is shown in Figure 1, where the longest reads correspond to Sanger reads and the mean length of GS-FLX Titanium reads are below the expected mean length due to the extremely high number of low quality nucleotides (52.5%, Table 2). SSH libraries account for the small shoulder on 200 bp in Figure 1A for Sanger reads.

### 2.4 Annotation

The annotation of pine sequences is especially challenging since, phylogenetically, pine is distantly related to angiosperm plant models, for which significantly more data and tools are already available [26]. EuroPineDB contains detailed and reliable annotations on UniGenes. This was achieved by combining the results of several annotation processes (described below). The redundant bioinformatics approach makes the curation and annotation processes highly reliable. During every EuroPineDB update, new sequences and new contigs will be re-annotated to provide the correct link and annotation as knowledge increases. Each sequence and UniGene has a specific page to display its annotation together with the *E *value associated with it to enable the empirical assessment of annotation quality. The current version of EuroPineDB includes annotations for 59.5% of pine UniGenes.

#### 2.4.1 Putative description

A sequence description is a user-friendly manner to offer information about putative functions. Every Sanger sequence in EuroPineDB is given a definition from up to four different sources, with the advantage that inconsistent descriptions are evidence of misannotation. Descriptions were obtained from: (i) the original description, if the sequence has one in the EMBL; (ii) the user definition, provided by the sequence owner when downloading sequences; (iii) the description retrieved by Blast2GO (see below); and (iv) the PGI definition obtained by the best hit in a low stringency BLAST (*E *< 10^-3^). Each UniGene contains only the definition provided by Blast2GO (see below).

#### 2.4.2 GO terms, EC keys, KEGG maps, and interpro codes

Every UniGene sequence was annotated using Blast2GO [21] using the best 10 sequences providing hits of at least 150 nt with a threshold *E*-value of 10^-10 ^against the non-redundant GeneBank in order to remove spurious annotations. In addition, GO terms with experimental evidence codes were the most preferred, while computational evidence codes and codes inferred by curator were half-weighted for the final annotation; GO terms without biological evidence data or inferred from electronic annotation were discarded. This provided annotations with a high degree of confidence for UniGenes using an *E*-value for evidence codes of 10^-6^. A Ruby script was designed to assign the corresponding metabolic pathway (a KEGG map) to each EC key provided by Blast2GO. InterPro codes obtained from Blast2GO were included to add other high-valued annotations (such as functional sites, protein families or conserved domains) since it is an integrated documentation resource [27]. Entries without annotations are candidates for re-annotation with every database update.

#### 2.4.3 SSRs, SNPs and ORFs

Plant cDNAs contain a high frequency of polymorphisms whose main sources are single nucleotide polymorphisms (SNPs) and single sequence repeats (SSRs). They serve to build molecular markers that form an essential starting point for association studies and other genome scan applications such as comparative genomics. SNPs and SSRs can also be used as templates to design primers that amplify specific genomic DNA in diverse populations [28]. SSRs have been assessed with MREPS (http://bioinfo.lifl.fr/mreps/ [29]). SNPs have been calculated with an adapted version of AlignMiner [28]. The tentative (complete or incomplete) ORFs were inferred from the results of Full-Lengther [30].

## 3 Utility and discussion

Molecular sequence databases are fundamental resources for modern bioscientists. The development of such a genomic resource for *Pinus pinaster *should facilitate basic and applied research on the genetics and evolution of this species, its role in maintaining forest health and ecosystem function, and the genetic traits that are desirable for the paper pulp and wood industry.

### 3.1 Web interface

**Figure 2:**
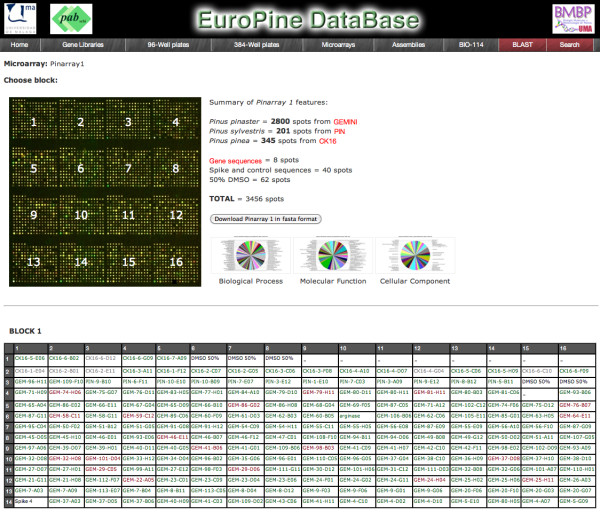
**An example of microarray page in EuroPineDB Web**. The upper part contains general information about the microarray as well as some statistical representation of the GO term distribution. The lower part is a representation of all sequences printed in a selected block. The colour codes are defined at the bottom of the Web page (not shown) and in the text.

EuroPineDB has been designed with a user-friendly interface (Figure 2) that can be browsed anonymously, although an authentication process has been considered to grant sequence owners the necessary permissions to browse their sequences privately, or browse through other authorised-while-private unpublished data. It has five top tabs and a menu on the left that enable database mining from different entry points. Two types of search tabs have also been implemented to perform queries and to retrieve and browse the resulting sequences. Information on tool versions and database releases used for obtaining the last update is shown on the right of the home page.

There is an option to download files containing UniGene sequences or sequences displayed on a page in FASTA format (including their QV when available), which facilitates further analyses by laboratories. Cross-links to the EMBL database are always provided by means of the accession numbers.

#### 3.1.1 Navigation tabs

By means of the 'Gene Libraries' tab, the user can see gene libraries, UniGenes and annotations for every gene library included in EuroPineDB. Each one contains a short description and some characteristics, including the statistical distribution of the relevant GO terms. The UniGene dataset consists of a consensus sequence of each contig and the singletons (see above).

Gene library clones are stored in 96-well and/or 384-well plates in the laboratory. Navigation using the '96-Well plates' and '384-Well plates' tabs displays the plate organisation of the libraries. Users can download the sequences of all clones in a plate or browse the plate in which red clones are useless sequences, green clones are those that have successfully passed SeqTrim pre-processing, and black ones are printed controls.

Currently, only one microarray (Pinarray1) has been designed with EuroPineDB sequences [22]. The 'Microarray' tab displays general and statistical information about Pinarray1 (Figure 2), whose printed sequences and annotations can be downloaded. Each microarray block organisation is displayed in the lower part of the page. Coordinates refer to a single sequence. The colours green, red and black have the same meaning as in the plates (see above). The graphic representation offers the possibility of retrieving information from specific clones after analysis of any experimental result using this microarray.

Sequences in EuroPineDB have been assembled by gene library and pine species, and can be accessed using the 'Assemblies' tab. Each assembly can be inspected in detail, showing a paged list of UniGenes and a summary description. The detailed view of every UniGene includes the aligned sequences, their orientation, the contig alignment (as a simple-text), a description for the consensus sequence, and the putative description of each included Sanger sequence.

**Figure 3:**
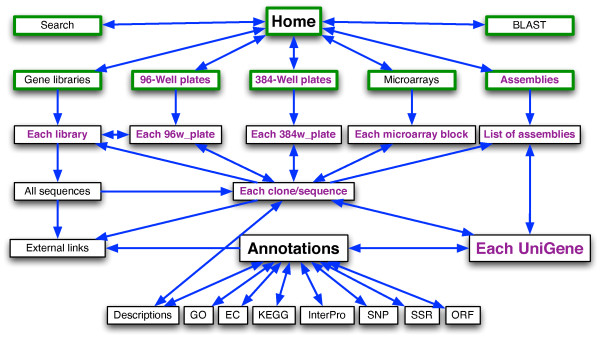
**Navigating through EuroPineDB**. Arrowheads indicate the direction of navigation. Green boxes correspond to available views from all pages (thus, no incoming arrowhead is specified). Violet text indicates the option of downloading sequences in FASTA format.

Clicking on the name of a clone provides access to all the information about it (e.g. EMBL accession number, sequence length, the plate(s) in which it can be found, annotations, original and pre-processed sequences, gene library source, etc). From the sequence entry, users can return to any previously described browsing page (Figure 3).

At the home page, a menu on the left enables filtered browsing by microarray, pine species, or annotation. Filtered browsing only displays entries sharing the same selected annotation. Each item in the list opens a new page with the EuroPineDB entries that share this specific annotation. For example, based on nitrogen metabolism (KEGG 00910), it is possible to know how many sequences are present in the database, since by clicking on 00910 every enzyme from this pathway can be seen, as well as the entries that are annotated as being one of these enzymes. As an additional example, all UniGenes involved in photosynthesis (GO:0015979) that belong to a particular library or pine species can be identified by means of GO term filtering.

#### 3.1.2 Database retrieval

In addition to a guided browsing, EuroPineDB contents can be retrieved by means of text search or sequence similarity. A text search engine has been implemented. It can look for words in annotations (i.e. descriptions for sequences, GOs, ECs and InterPro) or for specific codes (i.e. accession numbers or EuroPineDB identifiers). Search results can be restricted to different database subsets (displayed on the Web page as checkboxes), and they are then grouped by common characteristics and displayed in tabs which show/hide the list of elements that match the request. Results are also linked to their respective description pages.

A low-stringent (*E *< 10^-3^) BLAST-based search engine enables users to look for EuroPineDB entries similar to their amino acid or nucleotide sequence. The type of sequence (amino acid or nucleotides) is automatically detected, and either BLASTN or BLASTX will be used from the latest BLAST+ version [31]. BLAST searches may be conducted against different subsets of EuroPineDB: by species (*P. pinaster*, *P. sylvestris *and/or *P. pinea*, which can be chosen and combined as desired); and by single sequence or UniGenes. BLAST executions are queued and the results are accessible for up to 1 month with a custom URL that is sent to the user.

### 3.2 EuroPineDB is a large maritime pine sequence collection

EuroPineDB is mainly devoted to *P. pinaster *since its 877 523 curated reads (99.7% of total reads) have produced approximately 5.24 × 10^8 ^nt in 55 332 UniGenes (Table 2), 24 937 being > 500 bp. Assuming that a similar number of genes occur in *P. pinaster *as in *Arabidopsis thaliana *(25 000, which is close to the number of UniGenes > 500 bp) and a similar average transcript length (2000 nt), average transcriptome coverage was estimated at 10.5×. This amount of data and the high coverage represent a substantial sequence resource for *P. pinaster *that will contribute significantly to its genomic analysis and make EuroPineDB one of the largest sequence collections available for a pine species. Further evidence of the high coverage is that the number of *P. pinaster *UniGenes is slightly lower than the number of UniGenes in the Pp-454 library (55 431, Table 2). This indicates that UniGenes from the other gene libraries, which are also longer in size (Figure 1), have served to gather together apparently independent contigs from the 454 sequencing, and that most ESTs revealed by capillary (Sanger) analyses of cDNA libraries (Table 1) were also encountered in the 454-sequenced pooled RNA.

### 3.3 EuroPineDB sequences include a low occurrence of repetitive and retrotransposon-like sequences

The percentage of retrotransposon-like sequences in EuroPineDB is quite low (127 [0.0001%] reads and 20 [0.0003%] UniGenes), in contrast with the 6.2% found in *P. contorta*, indicating that mRNA isolation for cDNA synthesis in all gene libraries guarantees the best approach for gene discovery, instead of using total RNA. The reduced amount of repetitive DNA found in the coding sequences and the relatively long reads obtained by the sequencing procedures have enabled an accurate *de novo *assembly and a reliable UniGene collection of maritime pine. A relative high proportion of reads (876 839 out of 877 523, 99.92%) was assembled into reliable contigs (containing less than 9% mismatches), which is in agreement with other high-coverage assemblies [32].

### 3.4 EuroPineDB shed light on pine transcriptome

Since estimating the number of genes and the level of transcript coverage represented in an EST collection is an important issue for transcriptome sequencing projects, functional information of EuroPineDB UniGenes was included by means of the widely-used Blast2GO annotator [21]. A wide range of GO terms was assigned to EuroPineDB UniGenes indicating that a wide diversity of transcripts is represented in the database (results not shown). Therefore, 32 919 (59.5%) out of 55 332 UniGenes of *P. pinaster *were annotated and corresponded to at least 18 466 different genes (which is the number of unique UniProt hits). Assuming that UniGenes inferred from contigs longer than 500 bp are a reliable view of a transcriptome and observing that the number of *P. pinaster *UniGenes longer than 500 bp (20 928, including annotated and unannotated UniGenes) is slightly greater than the number of different UniGenes regarding unique UniProt hits (18 466), it can be inferred that much of the *P. pinaster *genes have been identified with the gene libraries described in Table 1, since both numbers (18 466 and 20 928) are close to the 25 000 genes that are supposed to form the *A. thaliana *genome.

The 59.5% of annotated maritime pine UniGenes is consistent with the 63.6% (20 928 out of 32 919 UniGenes) obtained when considering only UniGenes longer than 500 bp. Both percentages are only slightly lower than the 65% annotated in Eucawood [33] and the 67.8% in Melogen [34], but clearly more than the 32% of annotated sequences of *P. contorta *[2]. In total, 935 (1.7%) UniGenes were annotated with another pine sequence and 16 113 (29.1%) were annotated with a conifer (mainly *Picea*), which reflects the paucity of information on conifers in databases. This is further highlighted by the fact that the 12 057 (31%) annotated pine genes are qualified as "unknown" proteins, even though most of these unknown proteins correspond to non-annotated full-length transcripts from *Picea glauca *[35]. The predicted putative ORF could perhaps provide further support to any future functional annotation. The 40.5% of unannotated UniGenes may then correspond to one or more of the following possibilities: (i) putative new pine genes that do not have an orthologue; (ii) non-coding RNAs (including pseudogenes, antisense transcripts, structured RNAs, microRNAs, etc) that have recently been found in abundance when deep transcriptome analysis is performed [36,37]; (iii) short sequences from the UTR part that are difficult to match, even though 36.4% (11 991) of annotated UniGenes are shorter than 500 bp; and (iv) artefactual assemblies that do not correspond to valid protein-coding sequences, as occurs in 15% of entries in the human gene catalogue [38].

Each UniGene also includes information about putative SSRs and SNPs, since the development of SSR and SNP markers in pine species could serve to dissect complex traits given that linkage disequilibrium is low or declines rapidly within the length of an average-sized gene [39]. A total of 4740 SSRs have been found in EuroPineDB for *P. pinaster*; tri-nucleotide repeats were found to be the most common SSRs in EuroPineDB (55.7%), with tetra-nucleotide (12.6%) and di-nucleotide (10.5%) repeats being present at much smaller frequencies. This contrasts with *P. contorta *in which di-nucleotide repeats were the most abundant. A total of 44 185 SNPs were also identified. Most SSRs and SNPs (3546 [74.8%] and 41 152 [93.1%], respectively) occur in UniGenes with a putative ORF (2966 and 10 756, respectively), and 1% occurs in start/end codons. These numbers are difficult to compare to other conifers because of the different algorithms used for detection [2,18,39]. Due to the enormous size of the pine genome, ORF-based SSRs and SNPs are advantageous since they will reduce the mapping efforts required for the development of high-density maps and association studies. The development of SSR/SNP molecular markers, as well as the ORF predictions contained in EuroPineDB, will facilitate comparative genomics with other well-known conifers like *P. taeda, P. contorta*, *P. glauca *and *P. sitchensis*, and will be very useful to scientists interested in different aspects of pine genomics. In contrast to other plant databases, the SSRs and SNPs included in EuroPineDB are downloadable and can be used within any research project.

### 3.5 EuroPineDB differential features

EuroPineDB is a dynamic structure since its content is re-assembled and re-annotated when new sequences are added. It is designed to include new tables that display other pine genomic features in the near future. Its purpose is to make UniGenes and their annotations available to the scientific community involved in pine genomics by means of a flexible interface for developing queries. Although overlaps exist with the content of the Pine Gene Index (PGI) [16] and TreeGenes [17], each database offers distinct analytical approaches, enabling EuroPineDB to contain sequence relationships that cannot be found elsewhere. Moreover, EuroPineDB only includes sequences that have passed stringent quality filtering and only reliable annotations are assigned. Such a procedure provides a high level of confidence in the putative function and characteristics of *P. pinaster *UniGenes. Whereas the final aim of TreeGenes is to compare the different *Pinaceae *species, EuroPineDB, like ConiferEST [15], is more focused on deep information about a single species. In contrast to PGI and to ConiferEST, EuroPineDB differentiates pine species, contains the highest number of ESTs for a single conifer species, and provides insights on every gene library used to seed the database. ConiferEST attempted to provide reliable *P. taeda *EST pre-processing, anticipating the finding that at least 4.8% of ESTs in dbEST are contaminated by vectors, linkers, *E. coli *DNA and mitochondrial sequences [9]. TreeGenes does not fully pre-process chromatograms, since it was started using the high-quality sequence provided by PHRED; this has been described as a suboptimal strategy since it over-trims in relation to terminal structures, representing a loss of directional, positional and structural information on cDNA termini [20]. Since Pine Gene Index 7.0 extracted the sequences directly from databases, it contains untrimmed terminus parts, which has a detrimental impact on many downstream EST applications, thereby compromising the reliability of their resulting tentative contigs.

Early pre-processing of some gene libraries considered in EuroPineDB [40] have proven to be incorrectly processed and annotated (e.g. accession numbers BX252344, BX255382, BX252627, BX252630 or BX251344), and this is mainly due to using the now-outdated StackPack [41] workflows based on PHRED and PHRAP algorithms. EuroPineDB was pre-processed with SeqTrim [20] and assembled using CAP3 [23] and MIRA3 [25]. The use of SeqTrim was advantageous in obtaining reliable trimmed sequences, which is preferable to tailor-made scripts for every kind of sequence. An example of this improvement is indicated by the fact that EMBL v. 102 provided 12 673 out of 13 206 sequence entries (Table 2) devoid of any class of contaminant/artefactual sequences. Although this percentage could be considered too high for the EMBL database, it is clearly under the 4.8% reported for ESTs [9] because EMBL sequences have a more detailed curation process than ESTs. The use of SeqTrim is also devoid of the original reads of contaminating sequences from several bacteria and fungi genomes. Since contigs established with MIRA3 are highly reliable (the maximal mismatch percent in a contig is below 9%), EuroPineDB assemblies for *P. pinaster *would provide highly reliable UniGenes that would reflect a realistic set of pine genes.

## 4 Conclusion

EuroPineDB can be browsed intuitively (Figures 2 and 3) using several tabs, and data can be retrieved by text terms or sequence similarity using a stand-alone BLAST implementation. As a new feature, location information on sequences in microarrays is provided (Figure 2). UniGenes and its annotations can be browsed and downloaded (Figure 3) by pine species as well as by gene library, thus providing scientists with a comprehensive source of information on genomics and transcriptomics of *P. pinaster*. All this, together with the detailed sequence information and annotation, user-friendly Web-interface (Figure 2), regular updates, as well as connection to printed microarrays, make EuroPineDB extremely valuable to researchers using pine as a model organism, since its annotations and UniGenes cannot be found elsewhere. Finally, the current EuroPineDB assembly could also be used to design a new generation of pine microarrays comprising more UniGenes that would cover more transcriptome elements. Any scientists wishing to incorporate their sequences in EuroPineDB should contact the administrator to upload the data and obtain a private user account.

## Availability and requirements

Project name: EuroPineDB; Web site: http://www.scbi.uma.es/pindb; Operating system(s): platform independent; Programming language: Ruby, HTML; Other requirements: Ruby on Rails; Licence: e.g. GNU Affero Public License; Any restrictions to use by non-academics: licence needed.

## Conflict of interests statement

The authors declare that they have no competing interests.

## Authors' contributions

NFP analysed and annotated sequences, developed the database interface and put all information in the database. DGF designed and constructed the database and contributed to input/output scripting. JC, DPV, SDM, AFM, MAG, PP, CC, MTC and AS and RO conceived, designed and constructed the different gene libraries. AFM performed sequencing reactions. RB tested the functioning and created different case studies. FRC conceived and designed libraries and the microarray. RO, CA and FMC conceived and designed libraries and contributed to the interpretation of data. FRC and FMC were involved in drafting the manuscript. MGC conceived and designed the database, checked its functioning and wrote the manuscript. All authors read and approved the final manuscript.
